# What factors affect Beijing residents’ contracts with family doctors? A comparative study of Beijing’s urban and suburban areas

**DOI:** 10.3389/fpubh.2023.1159592

**Published:** 2023-07-06

**Authors:** Bo Lv, Chengsen Cui, Xingmiao Feng, Kai Meng

**Affiliations:** School of Public Health, Capital Medical University, Beijing, China

**Keywords:** family doctor contract services, influencing factors, districts, medical resources, China

## Abstract

**Objective:**

To improve the health of residents and promote hierarchical diagnosis and treatment to achieve an orderly pattern of medical treatment, Beijing implemented family doctor contract services (FDCSs) in 2011. The aims of this study were to analyze the current status of Beijing residents’ contracts with family doctors (FDs), compare the differences in contracting between urban and suburban residents, and explore the factors that affect residents’ contract behavior.

**Methods:**

From August 2020 to October 2020, a stratified sampling method was adopted to select residents from community health centers (CHCs) in districts D (urban area) and S (suburb) of Beijing to conduct a questionnaire survey. Chi-square tests, rank sum tests and logistic regression analyzes were used to analyze the current status and influencing factors of residents’ contracting with FDs.

**Results:**

A total of 4,113 valid questionnaires were included in the final analysis. District D was rich in medical resources, and the FD contract rate of residents there (93.09%) was significantly higher than that of residents in district S (78.06%; *p* < 0.05). Residents’ district (OR = 1.55, 95% CI = 1.18–2.05), understanding of FDCS policies (OR = 4.13, 95% CI = 3.63–4.69), preferred medical institutions (OR = 0.58, 95% CI = 0.42–0.79 for tertiary hospitals in the district; OR = 0.36, 95% CI = 0.22–0.59 for urban medical institutions in Beijing), age, education level, average annual medical expenses and medical insurance type were factors that influenced residents’ contracts with FDs (*p* < 0.05).

**Conclusion:**

This study shows that residents who are located in districts with rich medical resources, prefer CHCs as their first choice, have a better understanding of FDCS policies, and are more inclined to contract with FDs than other residents. It is recommended that the number and quality of FDs in suburban areas be increased and that medical staff strengthen publicity about FDCSs and actively encourage residents to contract with FDs.

## Introduction

Currently, many countries are becoming aging societies, the incidence of chronic noncommunicable diseases is on the rise, residents’ health needs are growing, and primary health care systems are challenged by an imbalance between supply and demand (1–3). Family doctor contract services (FDCSs) are important measures for improving the quality of primary health care and the health of residents (4–8). Several studies have demonstrated their effectiveness in encouraging residents to go to primary health care institutions for their first diagnosis as well as the control of medical costs (9–11).

There are differences in FDCS patterns and levels of development in different countries. The United Kingdom’s FDCS system is a major part of the National Health Service, and the government requires all residents to sign contracts with general practitioners (GPs). In the United States, GPs usually sign contracts with residents after the medical insurance company completes registration (12). Denmark has provided a prototype for FDCSs since 1973 and has essentially achieved full FDCS coverage (13, 14). However, the concept of a “family doctor” (FD) was introduced to China only in the late 1980s (15). China’s medical service system is mainly composed of hospitals and community health centers (CHCs). CHCs primarily provide community residents with diagnosis and treatment services for common and frequent illnesses as well as preventive and public health care. The Chinese FDCS is based on FD teams in CHCs and the principle of free choice by residents, providing residents with basic medical care, health management and public health services (16). In deepening the reform of the medical and health system in 2009, China proposed the goal of strengthening the capacity of primary medical services. The FDCS is an important measure to achieve this goal and was endowed with new connotations in this reform. After 10 years of exploration and development, China’s FDCS has created models such as Shanghai’s “1 + 1 + 1,” Xiamen’s “three divisions of comanagement,” and Hangzhou’s “family-based medical and nursing integrated services” (17). In 2011, Beijing began fully implementing the FDCS in 16 districts, requiring the homogenization of staff allocation for FD teams, service content and service modes.

The present study analyzed the factors that affect residents’ contracts with FDs from both qualitative and quantitative aspects. Yuan et al. and Xu et al. used qualitative research to show that the effect of implementing relevant policies, the government’s support for FDCSs and social capital affect residents’ contracts with FDs (18, 19). Other studies have used quantitative methods and found that the reimbursement rate of medical insurance, the medical level of CHCs, the chronic disease management needs of residents, satisfaction with FDs and awareness of FDCS policies are the factors that affect residents’ contracts with FDs (20–24). The allocation of medical resources may affect residents’ utilization of health services. The allocation of medical resources in urban areas is higher than that in suburban areas and high-quality medical resources are concentrated in hospitals, so urban residents are more willing to seek medical treatment in hospitals (25). With the improvement of medical resource allocation in hospitals, the probability of residents seeking medical treatment increases while the probability of residents seeking medical treatment in CHCs decreases, which may reduce the utilization of FDCSs (26). Higher allocation of medical resources may lead to a decrease in the probability of residents signing contracts with FDs. However, according to the above literature review, no research has explored the impact of the difference in the allocation of medical resources in different regions on the signing of contracts between residents and FDs. Therefore, this study innovatively proposes and conducts an empirical study to demonstrate that the allocation of medical resources may affect contracts between residents and FDs.

Research conducted in the Pudong District of Shanghai shows that according to household registration locations, suburban residents’ rate of contracting with FDs is higher than that of urban residents. The relatively insufficient medical resources in the suburbs make it easier to implement FDCSs, which is one of the reasons for the high contract rate of FDs in the suburbs, but no relevant empirical research has been conducted (27). There are differences in the allocation of medical resources between regions within different countries, especially between urban and rural areas in China (28–30). The allocation of medical resources in the urban area of Beijing is richer than in the suburbs (31), and access to health services for suburban residents is limited. Based on the above research results, we believe that the allocation of medical resources may affect the signing rate of FDs among Beijing residents. Residents in areas with rich medical resources are more willing to go to high-level hospitals for medical treatment, resulting in a low FD contracting rate. Residents in areas with insufficient medical resources tend to choose CHCs for treatment, so the signing rate of FDs is relatively high. To verify this hypothesis, residents of districts D (urban area) and S (suburban area) in Beijing were taken as the survey subjects. This allowed us to study the current situation and the factors that influence contracting between residents and FDs and to identify differences between the areas.

## Methods

### Study design

**Figure 1:**
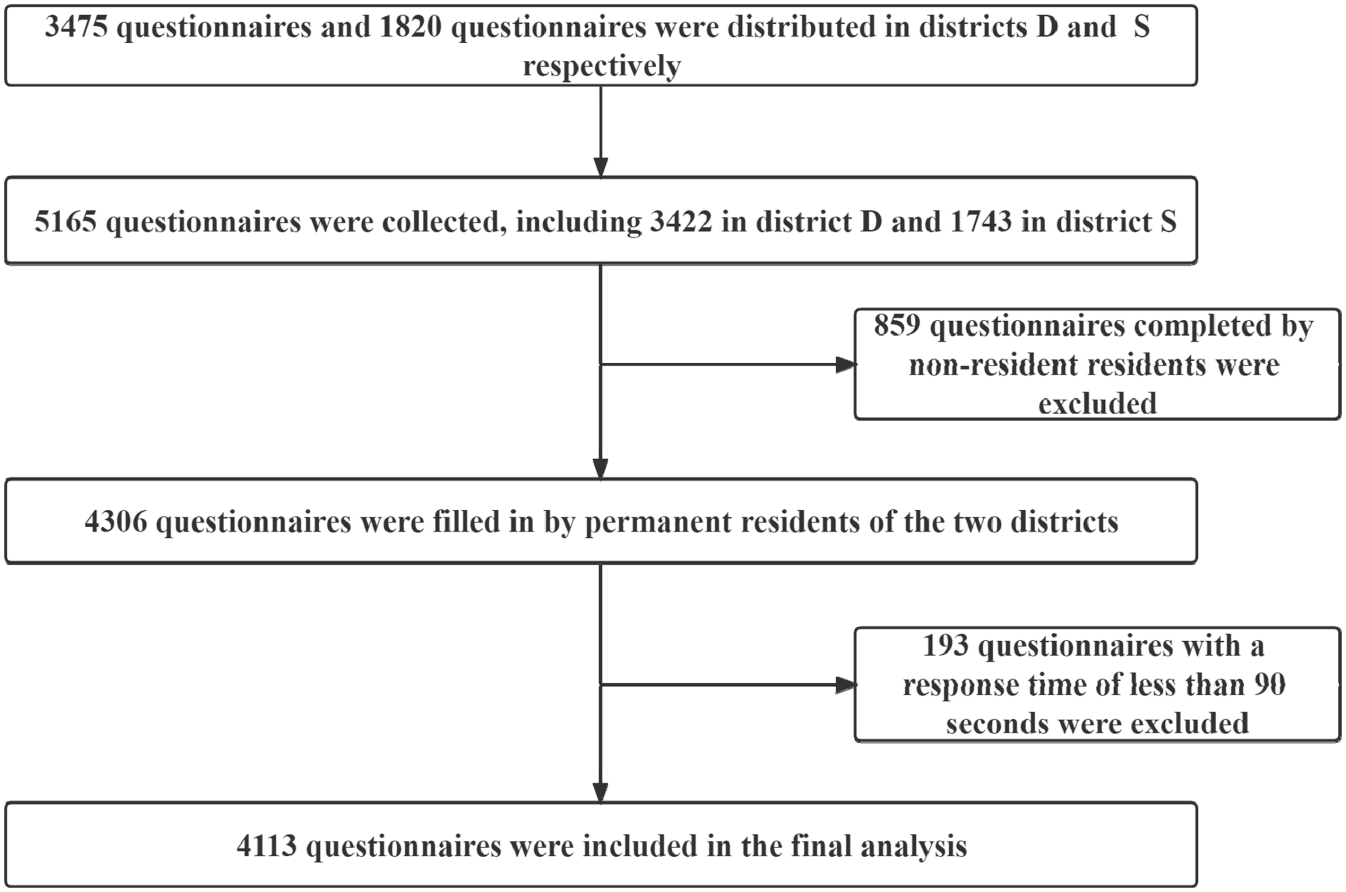
Sampling flow chart of this study.

The data come from a cross-sectional survey conducted in Beijing, China, in 2020. Beijing has 16 districts, and districts D and S were selected for this research from urban and suburban areas, respectively. There were great differences in economic level and medical resources between the two districts (see Table 1). Based on the outpatient volume of community health centers/stations in the two districts in 2019, residents were selected as the research subjects by a stratified sampling method. Due to the large difference in annual outpatient volume between community health centers/stations in districts D and S, it would not have been suitable to use the same standard to stratify community health centers/stations in the two districts and determine the number of residents to be investigated. Therefore, we determined the standard of stratified sampling separately for each of the two districts according to their annual outpatient volume of community health centers/stations. According to the annual outpatient volume of more than 50,000, 30,000-50,000, 10,000-30,000, and fewer than 10,000 person-times, the community health centers/stations in district D were divided into 4 categories. The sample sizes of each category were 100, 75, 50 and 25 people, respectively, and the total sample size was 3,475 people. According to the annual outpatient volume of more than 140,000, 100,000–140,000 and fewer than 100,000 person-times, the community health centers in district S were divided into 3 categories. The sample sizes of each category were 100, 70 and 50 people, respectively, with a total of 1,820 people, and the sample size of the two districts was 5,295 people. The inclusion criteria were as follows: permanent residents who were willing to participate in the study and who had the ability to make their own judgments. The exclusion criterion was as follows: after testing by the research group, the questionnaire completion time should be greater than 90 s. Thus, to ensure data quality, questionnaires with a response time of less than 90 s were excluded. Permanent residents of the two districts completed 4,306 questionnaires, and 193 questionnaires that did not meet the requirements were deleted. The final number of valid questionnaires was 4,113, and the effective response rate was 77.68%. The sample size selection process is shown in Figure 1.

**Table 1:** Basic characteristics of districts D and S.

Variable	District D	District S
Permanent population (ten thousand people)	79.4	122.8
Occupied area (km^2^)	41.8	1,021.0
GDP *per capita* (ten thousand RMB)	36.0	16.2
Number of health technicians per thousand people	32.7	7.4
Number of community health centers/stations	66.0	201.0
Number of tertiary hospitals	10.0	4.0

### Interviews and questionnaire

The questionnaire was designed based on a literature review and the results of focus group interviews. Through the literature review, we found that residents’ understanding of FDCS policies was the most prevalent and important factor that influenced residents to contract with FDs (32–36). Based on the number of people required for focus group interviews, this study conducted focus group interviews with FD team members in districts D and S and selected 6 FDs from CHCs for each interview (37, 38). A total of 12 family doctors participated in the interviews, including 1 nurse, 1 pharmacist, 2 assistant physicians, 5 attending physicians and 3 deputy chief physicians. The content of the interviews had two aspects: (1) What are the influencing factors of FDCSs? (2) How can FDCSs be promoted and improved? The results of the interviews were summarized separately to extract the potential influencing factors mentioned by the FDs. The two groups of FDs ranked the importance of the potential influencing factors separately, and the results showed that “preferred medical institution” was the most important potential influencing factor as perceived by both groups of FDs (39). Therefore, residents’ understanding of FDCS policies, preferences for medical institutions and basic information were investigated in the questionnaire.

The complete questionnaire consisted of two parts. The first part addressed the residents’ understanding and evaluation of FDCSs (28 questions), and the second part addressed the residents’ basic information (9 questions). The reliability and validity of the questionnaire in this study were good; the Cronbach’s α coefficient was 0.84, and the KMO value was 0.929 (*p* < 0.001; shown in Supplementary material). From August 2020 to October 2020, residents were surveyed through electronic questionnaires distributed to CHCs through the Health Commissions of districts D and S in Beijing.

### Measures

#### Dependent variable

For the question, “Are you currently contracting with FDs?,” answering “yes” meant that the respondent had contracted with FDs, and answering “no” or “I do not know” meant that the respondent had not contracted with FDs.

#### Independent variable

To verify the hypothesis of this research and combine the literature review and results of the focus group interviews, the residents’ district, the residents’ understanding of FDCS policies, and the residents’ preferred medical institution were used as independent variables: (1) The residents’ district was determined by the question, “What is your usual residence?” (2) The residents’ understanding of FDCS policies was measured by the question “Do you understand the relevant FDCS policies?” (3) The residents’ preferred medical institution was measured by the question, “If you are unwell (not critically ill), which medical institution do you generally prefer?”

#### Covariate variable

This study took the residents’ basic information as the covariate variable, which was assessed using the following questions: (1) “What is your gender?” (2) “What is your age?” (3) “What is your marital status?” (4) “What is your education level?” (5) “What is your average monthly income?” (6) “What are your average annual medical expenses?” (7) “What type of medical insurance do you have?” and (8) “What is your self-evaluation of your health?” All the variable assignments are shown in Table 2.

**Table 2:** Variable assignment.

Characteristic	Assignment
Contracting status	0 = Not signed; 1 = Signed
Resident district	0 = District S; 1 = District D
Understanding of FDCS policies	Very little understanding =1; Little understanding =2; General understanding =3; Some understanding = 4; High level of understanding =5
Preferred medical institution	
CHCs in the district	0 = No; 1 = Yes
First-level hospitals in the district	0 = No; 1 = Yes
Secondary hospitals in the district	0 = No; 1 = Yes
Tertiary hospitals in the district	0 = No; 1 = Yes
Urban medical institutions in Beijing	0 = No; 1 = Yes
Gender	0 = Male; 1 = Female
Age (years)	≤30 = 1; 31–40 = 2; 41–50 = 3; 51–60 = 4; ≥61 = 5
Level of education	High school or below = 1; Junior college = 2; Undergraduate = 3; Master’s degree or above = 4
Marital status	
Married	0 = No; 1 = Yes
Unmarried	0 = No; 1 = Yes
Divorced	0 = No; 1 = Yes
Widowed	0 = No; 1 = Yes
Average monthly income (yuan)	<2,000 = 1; 2,000–3,999 = 2; 4,000–5,999 = 3; 6,000–7,999 = 4; ≥8,000 = 5
Average annual medical expenses (yuan)	<1,000 = 1; 1,000–4,999 = 2; 5,000–8,999 = 3; 9,000–12,999 = 4; ≥13,000 = 5
Type of medical insurance	
UEBMI	0 = No; 1 = Yes
URBMI	0 = No; 1 = Yes
NMI	0 = No; 1 = Yes
CI	0 = No; 1 = Yes
Self-evaluation of health	Very good =1; Good =2; General =3; Poor =4; Very poor =5

UEBMI, Urban employee-based medical insurance; URBMI, Urban resident-based medical insurance; NMI, National medical insurance; CI, Commercial insurance.

#### Data analysis

IBM SPSS Statistics 26.0 was used for statistical analysis. The chi-square test was used to analyze gender, marital status, type of medical insurance, means by which residents learned about FDCS policies, preferred medical institutions, and contracting status. The rank sum test was used to analyze age, level of education, average monthly income, average annual medical expenses, self-evaluation of health, and residents’ understanding of FDCS policies. A logistic regression model was established to analyze the influencing factors of residents’ signing with FDs. Model 1 included residents’ basic information, Model 2 included residents’ preferred medical institution, Model 3 included residents’ understanding of FDCS policies, and Model 4 included residents’ districts. *p* < 0.05 indicated that the difference was statistically significant.

#### Ethics statement

This study was approved by the Ethics Committee of Capital Medical University. The survey was voluntary, and residents could refuse to participate. Informed written consent was obtained from all the participants before the start of this study. By completing a consent form, the participants were informed about the purpose and method of the study.

## Results

### Respondents’ basic information

Among all respondents in the two districts, the proportion of women was significantly higher than that of men, and people over 60 years old accounted for the largest group. The residents’ educational level was mainly concentrated at high school or below. The average monthly incomes of residents in districts D and S were concentrated at 4,000–5,999 yuan (1,442 people, 35.06%) and 2,000–3,999 yuan (537 people, 34.65%), respectively. The average annual medical expenses of residents were concentrated at 1,000-5,000 yuan. Most residents had urban employee-based medical insurance. Finally, residents had a good overall evaluation of their health. The characteristics of the respondents are presented in Table 3.

**Table 3:** Basic demographic characteristics of residents in districts D and S.

	Total *n* (%) (*n* = 4,113)	District D *n* (%) (*n* = 2,563)	District S *n* (%) (*n* = 1,550)	*χ* ^2^	*P*
Gender				1.98	0.085
Male	1,494 (36.32)	952 (37.14)	542 (34.97)		
Female	2,619 (63.68)	1,611 (62.86)	1,008 (65.03)		
Age (years)				−25.42	<0.001
≤30	319 (7.76)	98 (3.82)	221 (14.26)		
31–40	664 (16.14)	301 (11.74)	363 (24.42)		
41–50	699 (16.99)	305 (11.90)	394 (25.42)		
51–60	904 (21.98)	551 (21.50)	353 (22.77)		
≥61	1,527 (37.13)	1,308 (51.03)	219 (14.13)		
Level of education				−0.82	<0.001
High school or below	1,975 (48.02)	1,254 (48.93)	721 (46.52)		
Junior college	1,024 (24.90)	623 (24.31)	401 (25.87)		
Undergraduate	992 (24.12)	583 (22.75)	409 (26.39)		
Master’s degree or above	122 (2.97)	103 (4.02)	19 (1.23)		
Marital status				64.88	<0.001
Married	3,593 (87.36)	2,263 (88.29)	1,330 (85.81)		
Unmarried	284 (6.90)	122 (4.76)	162 (10.45)		
Divorced	111 (2.70)	79 (3.08)	32 (2.06)		
Widowed	125(3.04)	99 (3.86)	26 (1.68)		
Average monthly income (yuan)				−12.66	<0.001
<2000	263 (6.39)	69 (2.69)	194 (12.52)		
2000–3,999	1,061 (25.80)	524 (20.44)	537 (34.65)		
4,000–5,999	1,442 (35.06)	1,063 (41.47)	379 (24.45)		
6,000–7,999	678 (16.48)	452 (17.64)	226 (14.58)		
≥8,000	669 (16.27)	455 (17.75)	214 (13.81)		
Average annual medical expenses (yuan)				−13.62	<0.001
<1,000	844 (20.52)	376 (14.67)	468 (30.19)		
1,000–4,999	2,033 (49.43)	1,275 (44.75)	758 (48.90)		
5,000–8,999	645 (15.68)	448 (17.48)	197 (12.71)		
9,000–12,999	298 (7.25)	224 (8.74)	74 (4.77)		
≥13,000	293 (7.12)	240 (9.36)	53 (3.42)		
Type of medical insurance				256.68	<0.001
UEBMI	3,072 (74.69)	2,089 (81.51)	983 (63.42)		
URBMI	737 (17.92)	267 (10.42)	470 (30.32)		
NMI	260 (6.32)	184 (7.18)	76 (4.90)		
CI	44 (1.07)	23 (0.90)	21 (1.35)		
Self-evaluation of health				−6.81	<0.001
Very good	671 (16.31)	379 (14.79)	292 (18.84)		
Good	1,585 (38.54)	918 (35.82)	667 (43.03)		
General	1,590 (38.66)	1,079 (42.10)	511 (32.97)		
Poor	226 (5.49)	159 (6.20)	67 (4.32)		
Very poor	41 (1.00)	28 (1.09)	13 (0.84)		

UEBMI, Urban employee-based medical insurance; URBMI, Urban resident-based medical insurance; NMI, National medical insurance; CI, Commercial insurance.

### Status of contracts signed between residents and FDs

Table 4 shows the results, the first choice of 66.62% of the residents was CHCs when they were ill. By district, 1,985 residents in district D (77.45%) and 755 in district S (48.71%) indicated this preference. The proportion of residents in district D visiting CHCs was much higher than that in district S, indicating that residents in district D tended to go to CHCs for medical treatment when they were ill.

**Table 4:** Status of residents’ signing of contracts with FDs, understanding of FDCS policies and preferred medical institutions.

		Total *n* (%) (*n* = 4,113)	District D *n* (%)(*n* = 2,563)	District S *n* (%) (*n* = 1,550)	χ^2^	*P*
Residents’ signing of contracts with FDs	Contracting status				454.31	<0.001
	Signed	3,596 (87.43)	2,386 (93.09)	1,210 (78.06)		
	Not signed	517 (12.57)	177 (6.91)	340 (21.94)		
Residents’ understanding of FDCS policies	Residents’ understanding of FDCS policies				−14.95	<0.001
	Very little understanding	74 (1.06)	23 (0.90)	51 (3.29)		
	Little understanding	248 (6.03)	104 (4.06)	144 (9.29)		
	General understanding	532 (12.93)	246 (9.60)	286 (18.45)		
	Some understanding	1,691 (41.11)	1,025 (39.99)	666 (42.97)		
	High level of understanding	1,568 (38.12)	1,165 (45.45)	403 (26.00)		
	Means by which residents learned about FDCS policies				37.45	<0.001
	Medical staff introduction	3,059 (80.69)	2,027 (83.21)	1,032 (76.16)		
	Bulletin board or leaflet	285 (7.52)	142 (5.83)	143 (10.55)		
	Television, radio, internet, newspapers and other media	235 (6.20)	135 (5.54)	100 (7.38)		
	Introduction by family or friends	172 (4.54)	110 (4.52)	62 (4.58)		
	Other	40 (1.06)	22 (0.90)	18 (1.33)		
Residents’ preferred medical institution	Preferred medical institution				198.53	<0.001
	CHCs in the district	2,740 (66.62)	1,985 (77.45)	755 (48.71)		
	First-level hospitals in the district	565 (13.74)	153 (5.97)	412 (26.58)		
	Secondary hospitals in the district	152 (3.70)	70 (2.73)	82 (5.29)		
	Tertiary hospitals in the district	492 (11.96)	266 (10.38)	226 (14.58)		
	Urban medical institutions in Beijing	164 (3.99)	89 (3.47)	75 (4.84)		

FDs, Family doctors; FDCS, Family doctor contract service; CHCs, Community health centers. Some participants chose “Very little understanding” or “Little understanding,” and there was no need to answer the question “How did you learn about the FDCS policies?”; thus, the number of people listed in “Means by which residents learned about FDCS policies” is less than the total number of participants.

There was a statistically significant difference in the degrees of understanding FDCS policies and means of learning about them between the residents of districts D and S (*p* < 0.05). Residents had a better understanding of FDCS policies, and the proportion who chose “general understanding,” “some understanding” and “high level of understanding” was 92.27%. Among these, 2,027 people (83.21%) and 1,032 people (76.16%) in districts D and S, respectively, knew of FDCS policies mainly through publicity by medical staff.

In the overall survey responses, 87.43% of the residents had contracted with FDs; 93.09 and 78.06% of the residents in districts D and S, respectively, had done so. There was a significant difference in the residents’ contracting with FDs in the two districts (*p* < 0.05).

### Analysis of influencing factors of residents’ FD contracts

Table 5 shows the results, to explore the factors that influenced residents’ contracts with FDs, the variables were assessed using four logistic regression models. From Model 1 to Model 4, residents’ ages and average annual medical expenses were statistically significant. From Model 2 to Model 4, residents who preferred local CHCs were more willing to sign contracts with FDs. In Model 3 and Model 4, residents who knew more about FDCS policies were more inclined to contract with FDs. In Model 4, the residents’ district affected their signing with FDs. The R^2^ values of the four models gradually increased from 0.185 to 0.483, and the *p* values were less than 0.001. The Hosmer–Lemeshow test was performed on Models 1–4, and the *p* values were all greater than 0.05, indicating that the fit of the models was good.

**Table 5:** Analysis of factors affecting the behavior of residents signing with FDs (OR, 95% CI; *n* = 4,113).

Variable	Model 1	Model 2	Model 3	Model 4
Gender (Male as reference)				
Female	1.11 (0.90–1.36)	1.14 (0.92–1.41)	0.96 (0.75–1.24)	0.97 (0.76–1.25)
Age (years)	1.92 (1.73–2.12)^*^	1.83 (1.66-2.03)^*^	1.81 (1.61-2.04)^*^	1.68 (1.48-1.91)^*^
Level of education	0.93 (0.80–1.07)	0.91 (0.79–1.06)	0.81 (0.68–0.96)^*^	0.78 (0.65-0.92)^*^
Marital status (Married as reference)				
Unmarried	1.13 (0.82–1.56)	1.10 (0.79–1.53)	1.21 (0.81–1.80)	1.13 (0.76–1.69)
Divorced	1.28 (0.66–2.46)	1.24 (0.64–2.42)	1.51 (0.66–3.45)	1.45 (0.64–3.31)
Widowed	0.91 (0.41–2.00)	0.87 (0.39–1.95)	0.63 (0.27–1.46)	0.62 (0.26–1.44)
Average monthly income (yuan)	1.10 (0.99–1.22)	1.08 (0.97–1.20)	1.04 (0.92–1.18)	1.02 (0.90–1.15)
Average annual medical expenses (yuan)	1.42 (1.26–1.60)^*^	1.41 (1.25-1.59)^*^	1.33 (1.16-1.52)^*^	1.31 (1.15-1.51)^*^
Type of medical insurance (UEBMI as reference)				
URBMI	0.82 (0.62–1.08)	0.86 (0.65–1.13)	0.92 (0.67–1.28)	0.98 (0.71–1.36)
NMI	0.42 (0.29–0.61)^*^	0.48 (0.33-0.70)^*^	0.55 (0.36-0.85)^*^	0.56 (0.36-0.86)^*^
CI	0.35 (0.17-0.71)^*^	0.35 (0.17-0.75)^*^	0.72 (0.27-1.91)	0.73 (0.27–1.96)
Self-evaluation of health	0.79 (0.70–0.90)^*^	0.84 (0.74-0.96)^*^	1.11 (0.95-1.30)	1.10 (0.94–1.29)
Preferred medical institution (CHCs in the district as reference)				
First-level hospitals in the district		0.89 (0.66–1.21)	1.14 (0.79–1.64)	1.28 (0.89–1.86)
Secondary hospitals in the district		0.32 (0.21–0.49)^*^	0.59 (0.37-0.96)^*^	0.64 (0.39-1.03)
Tertiary hospitals in the district		0.35 (0.27–0.45)^*^	0.57 (0.42-0.77)^*^	0.58 (0.42-0.79)^*^
Urban medical institutions in Beijing		0.23 (0.16–0.35)^*^	0.36 (0.22-0.58)^*^	0.36 (0.22-0.59)^*^
Understanding of FDCS policies			4.20 (3.70–4.77)^*^	4.13 (3.63-4.69)^*^
Resident district (District S as reference)				
District D				1.55 (1.18–2.05)^*^
*R*^2^	0.185	0.229	0.480	0.483
*P*	<0.001	<0.001	<0.001	<0.001

^*^Indicates *p* < 0.05 FDs, Family doctors; FDCS, Family doctor contract service; UEBMI, Urban employee-based medical insurance; URBMI, Urban resident-based medical insurance; NMI, National medical insurance; CI, Commercial insurance, CHCs, Community health centers.

## Discussion

This study analyzed the current status of residents’ signing of contracts with FDs. The influence of medical resource allocation on residents’ contracting with FDs was considered and verified. Overall, 87.43% of the residents had contracted with FDs. Residents’ district, understanding of FDCS policies, preferred medical institution, education level, age, average annual medical expenses and type of medical insurance were factors that affected contracting between residents and FDs.

Residents in district D were more willing to sign contracts with FDs than residents in district S were. Since the allocation of medical resources in district S was relatively insufficient and the incidence of chronic diseases among suburban residents was higher than that among urban residents (40), FDCSs should have been more easily accepted by district S residents. However, the results of this study were contrary to our hypotheses. What caused the suburban FD contracting rate to be lower than the urban FD contracting rate? Most CHCs in districts D and S have established medical alliances with higher-level hospitals. However, the effects of medical alliances in districts with rich medical resources were better than those in districts with relatively insufficient medical resources; in addition, the medical service capacity of CHCs in the former was higher, and residents were more willing to go to such centers for treatment (41, 42). Well-known tertiary hospitals in district D serve patients from all over the country, and some residents prefer to go to high-level hospitals when suffering from common diseases (43). The high number of patients in hospitals caused residents to wait long times for treatment in tertiary hospitals, so they chose more convenient CHCs for treatment. District D was located in a central urban area with a higher economic level and more health knowledge among the public, and residents in this district had higher health needs and were more willing to sign contracts with FDs. Residents could obtain better service at CHCs in urban areas and were more willing to renew their contracts with FDs (44). Therefore, the FD signing rate among urban residents was higher than that among suburban residents.

District S had a relatively low economic level. Because of lower pay and fewer opportunities for career development, doctors with high educational backgrounds and professional titles prefer to work in urban areas (45, 46), and research has shown that most doctors in suburban areas do not have a bachelor’s degree (47), which increases residents’ distrust of doctors and reduces the FD contracting rate. To solve health problems through high-level medical services, suburban residents were more willing to go to high-level hospitals (48). In addition, the leverage effect of China’s medical insurance system on hierarchical diagnosis and treatment was not obvious, and it had little influence on residents’ choice of medical treatment. Moreover, the system lacked a mechanism design for FDCSs, which made them unattractive to residents (49), so residents’ willingness to contract with FDs decreased.

The factor that had the greatest impact on whether residents signed contracts was their degree of understanding of FDCS policies. Residents who knew more about FDCS policies were more inclined to sign contracts, which is the same conclusion drawn in Yuan’s (50) research in China. In the interviews, the FDs noted that “patients had a low awareness rate of the FDCS, and publicity efforts were not in place” and suggested that “the publicity efforts of the FDCS policies should be strengthened.” FDCS policies were most effectively publicized through medical staff, which suggests that they can increase FDCS publicity in the future. The medical staff of the CHCs established long-term relationships with residents, and some residents signed contracts with FDs following their introduction by doctors they already knew. In addition, tertiary medical institutions mainly treat patients with difficult and severe diseases, while CHCs mainly treat patients with chronic and common diseases; the latter can meet patients’ expectations in terms of treatment effects better than the former (51). Therefore, residents who preferred to go to CHCs were more willing to sign contracts with FDs.

It was difficult for older adult to obtain medicine from tertiary hospitals due to mobility limitations. After prescriptions are written by doctors in tertiary hospitals, older adult can receive medicine and treatment in CHCs. Older adult have a higher risk of illness, which may encourage them to pursue more targeted and continuous medical services (52). Therefore, the FD contracting rate among older adult is higher than that among individuals of other ages. Zhao’s (53) study showed that older adult with heart disease and diabetes have a higher demand for FDCSs. FDs can be more attentive to the health needs of the older adult population in these aspects and provide targeted services accordingly.

As their education level increases, individuals’ distrust in doctors increases, and the probability of preferring to go to CHCs to see doctors decreases (23). There are higher expectations for FDCSs, and a wait-and-see attitude is temporarily adopted among more highly educated individuals. The results of this study are similar to those of Shang and Jing (54, 55) but different from those of Huang (56). The impacts of education level on contracting between residents and FDs may differ due to differences in research objects, regions, and times. Further research on this subject is needed.

Residents suffering from chronic diseases have an economic burden, and the average annual medical expenses for these individuals are higher than those for residents without chronic diseases (57, 58). Chronic disease management, medication consultation and other services can improve the effects of chronic disease control and help individuals maintain their own health (59, 60), so residents with high average annual medical expenses were more willing to sign contracts with FDs. Compared with residents with UEBMI, residents with NMI were less willing to sign contracts with FDs. These residents are generally college students and public institution employees. Due to their better health, the signing rate of FDs in this population is relatively lower.

## Implications

FDCS is an important way to provide primary health care and a “gatekeeper” to control medical expenses, which plays an important role in countries around the world. This study found that residents in areas with good economic conditions and rich medical resources are more willing to sign contracts with FDs and are more willing to go to CHCs for their initial diagnosis. The main reason is determined by whether the medical service capacity of FDs can meet the medical service needs of residents. Improving the medical service ability of FDs is the main measure to increase the signing rate of FDs. The following measures are suggested. First, medical graduates with higher education should be recruited to carry out the FDCSs and introduce high-level doctors to work in CHCs. Second, the clinical skill training of FDs should be increased, and more career development opportunities should be provided. Third, the salary of FDs should be improved, and an effective incentive mechanism should be formulated. In addition, this study found that residents’ understanding of FDCS policies affects their signing with FDs. Personalized FDCS packages can be developed according to the needs of residents, and publicity of FDCS can be strengthened to let residents know the content of FDCS.

## Strengths and limitations

The merit of this study is its exploration of the differences in residents’ contracts with FDs in two districts with large differences in medical resources. Through the analysis, the influence of residents’ resident districts on FD contracting rates was verified. However, we acknowledge that this study has some limitations. First, the cross-sectional survey data used in this study should be interpreted as having an associative effect, so further longitudinal studies are needed. Second, this study only conducted investigations in Beijing. Beijing’s medical resources are better than those of other provinces, and the government invests more in CHCs in Beijing. The management and operation of CHCs are relatively good. Therefore, the conclusions of the study have certain limitations, and other regions can be examined for further advances in follow-up studies. Finally, there are many factors that may affect residents’ contracts with FDs. The factors included in this study may not be comprehensive, which can be further improved in subsequent studies.

## Conclusion

This study selected two districts in Beijing with relatively large differences in medical resources to examine the current status and influencing factors of contracts signed between residents and FDs. The analysis concluded that the allocation of medical resources affects residents’ signing of contracts with FDs. Residents in districts with rich medical resources were more willing to sign contracts with FDs than other residents were. The role of medical resource allocation should thus be considered in future research. Residents who preferred CHCs and knew more about FDCS policies were more willing to sign up with FDs. It is recommended that with the support of the government, FD service ability should be improved and the publicity of FDCSs should be strengthened. More policy incentives and financial support should be given to areas with relatively insufficient medical resources, increasing the rate of FD contracts.

## Data availability statement

The raw data supporting the conclusions of this article will be made available by the authors, without undue reservation.

## Ethics statement

The studies involving human participants were reviewed and approved by This study was approved by the Ethics Committee of Capital Medical University. Our survey was voluntary, and residents could refuse to participate. Informed written consent was obtained from all the participants before the start of this study. By completing a consent form, the participants were informed about the purpose and method of the study. The patients/participants provided their written informed consent to participate in this study.

## Author contributions

BL: methodology, formal analysis, data curation, and writing–original draft preparation. CC: investigation, data curation, and writing–review and editing. XF: writing–review and editing. KM: conceptualization, writing–review and editing, supervision, and funding acquisition. All authors contributed to the article and approved the submitted version.

## Funding

This study was supported by the National Natural Science Foundation of China (72274128). The funders had no role in any aspects of this study, including the study design, data collection and analysis, decision to publish or preparation of the manuscript.

## Conflict of interest

The authors declare that the research was conducted in the absence of any commercial or financial relationships that could be construed as a potential conflict of interest.

## Publisher’s note

All claims expressed in this article are solely those of the authors and do not necessarily represent those of their affiliated organizations, or those of the publisher, the editors and the reviewers. Any product that may be evaluated in this article, or claim that may be made by its manufacturer, is not guaranteed or endorsed by the publisher.

## Abbreviations

FDCS: family doctor contract service
CHC: community health center
FD: family doctor
UEBMI: Urban employee-based medical insurance
URBMI: Urban resident-based medical insurance
NMI: National medical insurance
CI: Commercial insurance
GP: General practitioner
