# Supplementary material for: What factors affect Beijing residents’ contracts with family doctors? A comparative study of Beijing’s urban and suburban areas
Source: Front Public Health. 2023 Jul 6;11:1159592. doi: 10.3389/fpubh.2023.1159592 (PMC10356989; doi:10.3389/fpubh.2023.1159592)
Supplement: Supplementary file 1 [file Data_Sheet_1.PDF]

Family doctor contract service questionnaire

Dear Sir/Madam,

Hello! Thank you for taking time out of your busy schedule to complete this survey! At present, we are carrying out a questionnaire survey on the family doctor contract service. The purpose is to understand your utilization of family doctor contract service, so as to provide a scientific basis for improving quality of family doctor contract service.

This questionnaire is filled in anonymously, there is no right or wrong for you to answer, the survey results are only used for scientific research, please fill in according to your actual situation, your careful filling is very important for the research, we will strictly abide by the standards of research, your answer is strictly confidential.

Thank you for your support and cooperation!

I. Cognition and evaluation of family doctor contract service

1.If you feel unwell (non-critical), what is your first choice of medical institution?

A. CHCs in the district B. First-level hospitals in the district C. Secondary hospitals in the district D. Tertiary hospitals in the district E.Urban medical institutions in Beijing

2. What is your reason for choosing this institution? (three choices only)

A. Convenient proximity to home B. Low cost of medical care C. Good level of medical care D. Good service attitude E. Good medical environment F. acquaintances G. Medical insurance fixed point H. Complete medicines I. Other ___________

3. Do you think the doctors in your community are capable of treating common diseases?

A. Completely unavailable B. Basically unavailable C. Generally available D. Basically available E. Completely available

4. Do you know the relevant policies of family doctor contract service? (Select A/B to jump to question 6)

A. Very little understanding B. Little understanding C. General understanding D. Some understanding E. High level of understanding

5. You know the source of family doctor contract service policy:_______________

A. Medical staff introduction B. Billboard or leaflet C. TV, radio, internet, newspaper and

other media D. Introduction of family and friends E. Others_______________

6. Have you signed a family doctor at present?

A. Yes B. No C. Not clear

The following are the issues of contracted residents:

7. What is the main reason for you to sign up for family doctors? (Select only three items)

A. Introduction by familiar doctors B. Convenient to see a doctor and take medicine C. Cheap to see a doctor D. Consult medical problems E. Follow up patients with chronic diseases regularly F. Provide door-to-door services G. Provision of appointment/referral services H. Government regulations I. Others_______

8. Do you know the service content of family doctor team? (including health archives, health management, health promotion and other services)

A. Very little understanding B. Little understanding C. General understanding D. Some understanding E. High level of understanding

9. Have you ever received family doctor team services (excluding daily diagnosis and treatment services) since you signed the contract? (Select B/C to jump to question 12)

A. Yes B. No C. Not clear

10. What family doctor team services have you received since signing the contract? (Multiple choices are allowed)

A. Family bed B. Health consultation C. On-site medical service D. Rehabilitation guidance E. Medication guidance F. Appointment registration, referral G. Chronic disease management H. "Prevention of disease" service of traditional Chinese medicine I. Regular measurement of blood pressure, blood sugar J. Physical examination K. Health management of the elderly, women, children and other key groups L. Nutritional diet guidance M. Psychological counseling N. Establishment of health records O. Others____________

11. Is the family doctor contract service effective for your disease improvement or health maintenance?

A. Not at all B. Not at all C. General D. More useful E. Very useful

12. Are you willing to continue signing next year? (If you choose A, go to question 14)

A. Yes B. No C. Not sure

13. The reason why you are not willing to renew your contract is_______________ (Select only three items)

A. No need B. Concerned about the impact of contracted family doctors on free medical treatment C. The service level of family doctors is not enough D. The type and quantity of drugs are small. E. Seldom come to the community for medical treatment at ordinary times. F. Increase medical expenses G. Others____

14. Are you willing to recommend your family and friends to sign a family doctor?

A. Very unwilling B. Not very willing C. General D. More willing E. Very willing

15. Are you satisfied with the medical level of family doctors?

A. Not satisfied B. Not very satisfied C. General D. Quite satisfied E. Very satisfied

16. Are you satisfied with the examination equipment and drug supply of the family doctor?

A. Not satisfied B. Not very satisfied C. General D. Quite satisfied E. Very satisfied

17. Are you satisfied with the service attitude of family doctors?

A. Not satisfied B. Not very satisfied C. General D. Quite satisfied E. Very satisfied

18. Are you satisfied with the services of family doctors?

A. Not satisfied B. Not very satisfied C. General D. Quite satisfied E. Very satisfied

19. Your evaluation on the overall service of family doctors:_______________

A. Not satisfied B. Not very satisfied C. General D. Quite satisfied E. Very satisfied

20. Your support for family doctor policy:_______________

A. Not supported B. Not very supported C. General D. More supported E. Very supported

The following are the issues of non-contracting residents:

7. Why did you not sign a family doctor?

A. Don't know B. Don't need C. The type and quantity of drugs are small D. Concern about the cost E. The medical level of family doctors is not as good as that of doctors in large hospitals. F. Concerned about signing family doctors and affecting free medical treatment

8. Are you willing to sign a family doctor in the future?

A. Very unwilling B. Not very willing C. General D. More willing E. Very willing

9. Are you satisfied with the medical level of the institution?

A. Not satisfied B. Not very satisfied C. General D. Quite satisfied E. Very satisfied

10. Are you satisfied with the inspection equipment and drug supply of the institution?

A. Not satisfied B. Not very satisfied C. General D. Quite satisfied E. Very satisfied

11. Are you satisfied with the service?

A. Not satisfied B. Not very satisfied C. Average D.Fairly satisfied E. Very satisfied

12. Are you satisfied with the service items of the agency?

A. Not satisfied B. Not very satisfied C. General D. Quite satisfied E. Very satisfied

13. Your evaluation of the overall service of the institution:_______________

A. Not satisfied B. Not very satisfied C. General D. Quite satisfied E. Very satisfied

14. Your support for family doctor policy:_______________

A. Not supported B. Not very supported C. General D. More supported E. Very supported

Ii. Basic information

1.Your gender: ________A. Male B. Female

2. Your age is: _________A.≤30 years old B. 31-40 years old C. 41-50 years old D. 51-60 years old E.≥61 years old

3. Your education level is: ____A. High school (technical secondary school) and below B. Junior college C. Bachelor degree D. Master degree or above

4. Your marital status: ________A. Married B. Unmarried C. Divorced D. Widowed

5. Your usual address is _____A. D district B. S district C. Others____

6. Your current average monthly income is: _______________

A. Less than 2000 yuan B.2000-3999 yuan C.4000-5999 yuan D.6000-7999 yuan E.8000 yuan and above

7. Your annual medical expenses (expenses paid by yourself except medical insurance) are: _______________ (including the sum of all expenses for outpatient service, hospitalization, physical examination and medicine purchase)

A. Less than 1000 yuan B.1000 yuan -5000 yuan C.5000 yuan -9000 yuan

D.9000 yuan - 13,000 yuan E. More than 13,000 yuan

8. Your main type of medical insurance is (fill in the main type of insurance) :

A. Urban employee-based medical insurance B. Urban resident-based medical insurance

C. National medical insurance   D. Commercial insurance

9. Your self-evaluation of health status is: _______________

A. Very good   B. Relatively good   C. General   D. Poor   E. Very poor
