# Supplementary material for: What factors affect Beijing residents’ contracts with family doctors? A comparative study of Beijing’s urban and suburban areas
Source: Front Public Health. 2023 Jul 6;11:1159592. doi: 10.3389/fpubh.2023.1159592 (PMC10356989; doi:10.3389/fpubh.2023.1159592)
Supplement: Supplementary file 2 [file Data_Sheet_2.PDF]

The reliability and validity of the questionnaire in this study were good; the Cronbach's α coefficient was 0.84, and the KMO value was 0.929 (P<0.001).

Table 1 Reliability statistics

Cronbach's α coefficient	Number of terms
0.840	9

Table 2 KMO and Bartlett tests

	statistic	value
	KMO sampling appropriateness measure	0.929
Bartlett's sphericity test	Approximate cardinality	23681.412
	Degrees of freedom	36
	Significance	＜0.001
